# Annexins in Glaucoma

**DOI:** 10.3390/ijms19041218

**Published:** 2018-04-17

**Authors:** Timothy E. Yap, Benjamin Michael Davis, Li Guo, Eduardo M. Normando, Maria Francesca Cordeiro

**Affiliations:** 1 The Western Eye Hospital, Imperial College Healthcare NHS Trust (ICHNT), London NW1 5QH, UK; timothyedward.yap@nhs.net (T.E.Y.); eduardomaria.normando1@nhs.net (E.M.N.); 2 The Imperial College Ophthalmic Research Group (ICORG), Imperial College, London NW1 5QH, UK; 3 Glaucoma and Retinal Neurodegeneration Group, Department of Visual Neuroscience, UCL Institute of Ophthalmology, London EC1V 9EL, UK; dr.bmdavis@gmail.com (B.M.D.); l.guo@ucl.ac.uk (L.G.)

**Keywords:** glaucoma, annexin, retinal ganglion cell, imaging, apoptosis, neurodegeneration

## Abstract

Glaucoma is one of the leading causes of irreversible visual loss, which has been estimated to affect 3.5% of those over 40 years old and projected to affect a total of 112 million people by 2040. Such a dramatic increase in affected patients demonstrates the need for continual improvement in the way we diagnose and treat this condition. Annexin A5 is a 36 kDa protein that is ubiquitously expressed in humans and is studied as an indicator of apoptosis in several fields. This molecule has a high calcium-dependent affinity for phosphatidylserine, a cell membrane phospholipid externalized to the outer cell membrane in early apoptosis. The DARC (Detection of Apoptosing Retinal Cells) project uses fluorescently-labelled annexin A5 to assess glaucomatous degeneration, the inherent process of which is the apoptosis of retinal ganglion cells. Furthermore, this project has conducted investigation of the retinal apoptosis in the neurodegenerative conditions of the eye and brain. In this present study, we summarized the use of annexin A5 as a marker of apoptosis in the eye. We also relayed the progress of the DARC project, developing real-time imaging of retinal ganglion cell apoptosis in vivo from the experimental models of disease and identifying mechanisms underlying neurodegeneration and its treatments, which has been applied to the first human clinical trials. DARC has potential as a biomarker in neurodegeneration, especially in the research of novel treatments, and could be a useful tool for the diagnosis and monitoring of glaucoma.

## 1. Glaucomatous Neurodegeneration and Challenges in Its Management

Glaucoma is a progressive, neurodegenerative disease of the optic nerve, which is characterized by retinal ganglion cell (RGC) apoptosis [1,2,3,4,5,6]. The loss of the retinal nerve fiber layer causes thinning of the neuroretinal rim and excavation of the optic nerve head, which is commonly described as “cupping” [7]. This loss of optic nerve function often leads to characteristic peripheral visual field defects, which can progress to defects in the central vision and can lead to complete blindness if not adequately treated in a timely manner [8]. Unfortunately, patients will often be asymptomatic due to the peripheral location of their visual field defects and “filling in”, therefore presenting late in the course of their disease [9,10].

**Figure 1:**
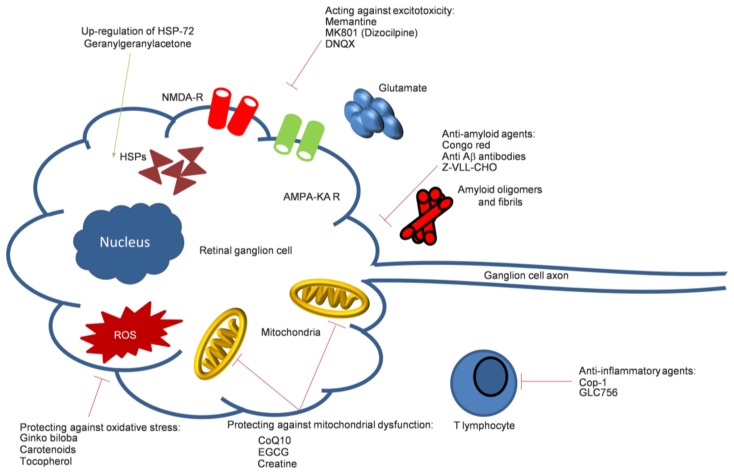
A diagrammatic summary of possible neurodegenerative mechanisms and their targeting (represented by line indicators, green: excitatory, red: inhibitory) in glaucomatous damage (Cop-1: Copolymer-1; CoQ10: Coenzyme Q10; DNQX: 6,7-Dinitroquinoxaline-2,3-dione; EGCG: Epigallocatechin galleate; Z-VLL-CHO: N-benzyloxycarbonyl-Val-Leu-leucine).

**Figure 2:**
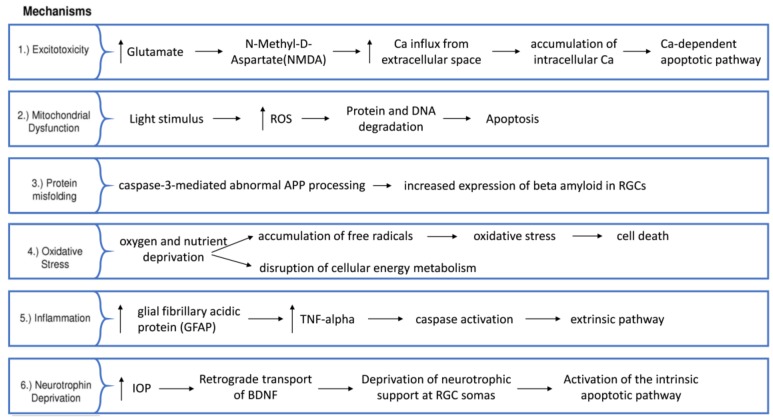
Glaucoma pathophysiological pathways (APP: amyloid precursor protein, upwards arrow: upregulation).

The most common subtype of glaucoma is primary open-angle glaucoma (POAG) [11]. Several risk factors have been identified, with intraocular pressure (IOP) being the only modifiable one. This is determined by a fine balance of aqueous humor production from the ciliary body and drainage through both the trabecular meshwork and the uveoscleral pathway [12]. If the resistance to drainage [12] causes an IOP that is high enough to risk further glaucomatous damage, pressure can be reduced with medical, laser and surgical therapy [13,14,15]. Other non-modifiable risk factors identified in glaucoma include increasing age [16], black race [17], family history [18] and myopia [19,20]. There are several theories hypothesized for the mechanism of RGC damage, with multiple disease variants being possible although they are still indistinguishable. The lamina cribrosa is the structure through which the optic nerve fibers penetrate the sclera of the eye and has been proposed as a site of nerve fiber vulnerability due to mechanical stress. Intraocular pressure has been suggested to cause damage to axons within the lamina, interrupting axonal transportation, which is known to occur early in experimental glaucoma and has been found in post-mortem specimens [21,22]. Another theory involves excitotoxicity, which implicates excess glutamate activity (the main excitatory neurotransmitter in the central nervous system) in the triggering of apoptosis [23,24]. In addition, other theories of vascular dysregulation [25], immunological factors [26] and oxidative stress [27,28] remain foci of interest for potential novel therapies (Figure 1 and Figure 2).

The current gold-standard investigation for diagnosis and monitoring is Standard Automated Perimetry (SAP), which assesses the patient’s peripheral field of vision [29,30]. SAP is carried out on each eye separately, which involves presenting targets to the patient, who then presses a button in response to its appearance at certain locations. This is mapped out for assessment by the clinician and for automated progression analysis [31,32].

Glaucoma imaging is currently dominated by optical coherence tomography (OCT) technology, which provides non-invasive cross-sectional imaging of the retina and automated segmentation of the retinal nerve fiber layer and ganglion cell complex. Single baseline images hold moderate value in predicting future glaucoma progression due to the heterogeneity of normal anatomical variants [33,34]. Monitoring serial progression in thinning of these anatomical structures of interest is more informative. However, during the time period required for detecting progression using both imaging and visual field testing, irreversible visual loss may have occurred [34,35,36].

Optimal glaucoma management involves early and accurate diagnosis [37]. This enables IOP-lowering treatment to be targeted to the patients with progressive disease. However, this is complicated by certain patients, who progress despite adequately treated IOP. The Detection of Apoptosing Retinal Cells (DARC) project has proposed an objective, pressure-independent method for detecting rates of glaucomatous degeneration in real-time, using fluorescently-labelled annexin A5 to quantify apoptosing RGCs.

## 2. In Vivo Apoptosis Quantification with Annexin A5

Apoptosis, or programmed cell death, is a process that was first described by Kerr et al. in 1972, which plays a central role in the normal development and regulation of multi-cellular organisms [38]. Alterations in the rate of apoptosis have been implicated in the pathology of many diseases, including different types of cancer and disorders of the central nervous system, some of which are summarized in Table 1. Apoptosis is characterized by sequential morphological and biochemical changes to cells that include shrinkage, blebbing of the membrane, chromatin condensation and the development of pyknotic nuclei [39]. One of the earliest signals for detecting apoptosing cells prior to the occurrence of gross morphological changes involves the presentation of the anionic phospholipid phosphatidylserine on the outer leaflet of the cell membrane, which was first reported by Fadok et al. in 1992 [40].

Under normal conditions, the distribution of phosphatidylserine is maintained in an asymmetric state by a family of ATP-dependent proteins called flippases [41], which predominantly reside in the inner leaflet of the cell membrane. On induction of apoptosis, the externalization of phosphatidylserine occurs as a result of flippase downregulation in conjunction with the activation of scramblase proteins, which induce the non-specific and bidirectional movement of phospholipids between the bilayer leaflets in a calcium-dependent fashion [42]. In apoptosis, the externalization of phosphatidylserine is thought to act as an “eat-me” signal, attracting phagocytes to engulf cells undergoing apoptosis [40].

Due to the large number of processes in which apoptosis dysregulation has been implicated, it is perhaps not surprising that many groups have sought to develop tools to monitor the extent of apoptosis both in vitro and in vivo. Annexin A5 is an endogenous 36 kDa protein ubiquitously expressed in humans that electrostatically binds to phosphatidylserine (and phosphatidylethanolamine to a lesser extent) in a calcium-dependent manner, with an affinity in the order of 10^−9^ M [57]. Although the biological function of annexin A5 is not fully understood, it is reported to play a role in the regulation of membrane permeability [58] and membrane repair [59], the promotion of autophagy by induction of autophagosome–lysosome fusion [60] and anti-endotoxin activity [61]. Furthermore, it may also act as a ligand for the complement protein C1q during apoptosis induction [62].

By exploiting the phosphatidylserine-binding property of annexin A5, fluorescently conjugated annexin A5 (fluorescein-annexin A5) was first proposed as an early stage marker of in vitro apoptosis using flow cytometry by Vermes et al. in 1995 [63]. Today, fluorescently labelled or biotinylated annexin A5 is routinely used in conjunction with propidium iodide (PI) as an in vitro assay to differentiate between apoptotic, necrotic and healthy cells for both flow cytometry [64] and imaging applications [65].

The first use of annexin in patients was with radiolabels, such as ^18^F, ^124^I or ^99m^Tc [66,67,68]. Technetium-99m annexin A5 was described by Ohtsuki et al. in 1999 for the non-invasive visualization of thymic apoptosis in rodents [69]. Dual radio- and fluorescently-labelled annexin A5 was utilized with in vivo single-photon emission computed tomography (SPECT) imaging of rodent models to investigate the macrophage infiltration of atherosclerotic plaques [70,71], tumor apoptosis visualization [72], prosthetic joint infection [73] and stroke [74] (although the latter is considered to be somewhat controversial [75]). More recently, the synthesis of annexin A5 functionalized nanoparticles has been described to permit multimodal imaging in rodents with computed tomography imaging of atherosclerotic plaques [76]. Furthermore, annexin A5-conjugated core-cross-linked polymeric micelles [77] and cross-linked iron oxide (CLIO) magnetic nanoparticles [78] have also been described. In humans, Technetium-99 m labelled annexin A5 has been featured in over 20 clinical trials, including monitoring tumor apoptosis in response to therapy [79], stroke severity [74] and prosthetic joint infection [80].

## 3. Retinal Apoptosis Visualization with Annexin A5

In contrast to most human tissues, the eye (specifically the cornea and lens) is optically transparent. This permits the use of techniques, such as confocal scanning laser ophthalmoscopy (cSLO) and optical coherence tomography (OCT) [81]. Unfortunately, due to their low contrast edges, RGCs are extremely difficult to visualize [82]. This is despite recent developments where a single RGC resolution is technically feasible. However, the small field of view, prolonged image acquisition and analysis time (to see only a few hundred RGCs of a typical population of >1 million cells) and requirement of adaptive optics (AO) functionality currently limits its clinical use [83].

**Figure 3:**
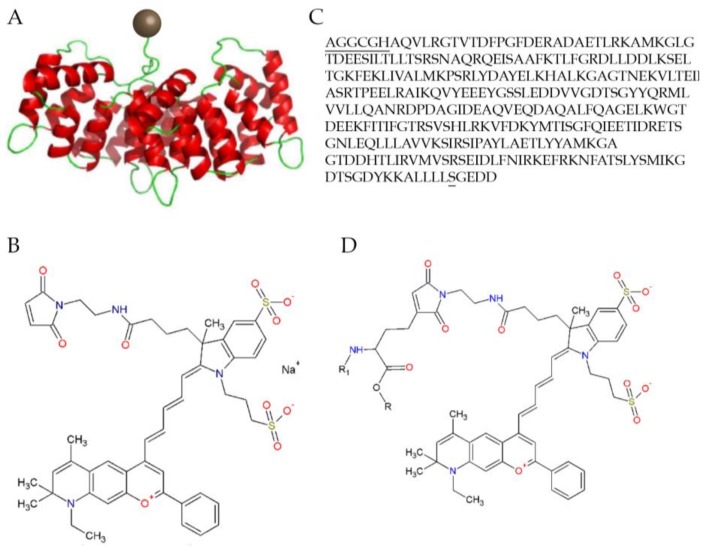
The structure of the Dy-776-mal labelled annexin V128 molecule: (**A**) Diagrammatic representation with the Dy-776-mal label represented with a brown sphere and the annexin molecule in red and green; (**B**) The molecular structure; (**C**) The optimized amino acid sequence of Anx V128, with the additional amino acids at the N-terminus containing the cysteine and mutated serine; and (**D**) The molecular structure of the fluorescent conjugation of ANX776 [85].

The DARC technique enables better visualization at a single cell resolution due to the presence of the fluorescent tag acting as a “contrast agent”. Two fluorescently-labelled annexin A5 molecules have been trialed thus far in DARC studies, including Alexa Fluor 488-labeled annexin A5 and a fluorescently-labelled variant of human annexin A5, RhAnnexin V128 (ANX776, see Figure 3). The former of these, Alexa Fluor 488-labeled annexin A5, has excitation/emission wavelengths of 495/519 nm and can be excited with an Argon laser at 488 nm. The latter, ANX776, uses the variant annexin molecule to facilitate a single covalent bond between the maleimide form of the Dy776 fluorescent dye and the cysteine residue of the RhAnnexin V128. This has excitation/emission wavelengths of 771/793 nm in the near-infrared region similar to indocyanine green (ICG) (a dye currently used in fundus fluorescence angiography to diagnose retinal conditions, such as idiopathic polypoidal choroidal vasculopathy [84]), enabling already widely-available clinical instruments to be used with DARC. The biological function of ANX776 was confirmed in vitro as it showed an affinity to phosphatidylserine in a calcium-dependent manner [85].

The imaging of the retina with DARC uses confocal scanning laser ophthalmoscopy, which is a high-resolution method of taking optical sections through biological tissues while having the advantage over conventional optical microscopy in terms of the depth of field selectivity to eliminate out-of-focus information [86]. The excitation of the fluorophores at their specific excitation wavelength occurs by laser illumination. After this, the reflected light of the emission wavelength of that fluorophore is filtered and acquired to highlight the uptake of the annexin molecule to the surface of apoptosing cells.

**Figure 4:**
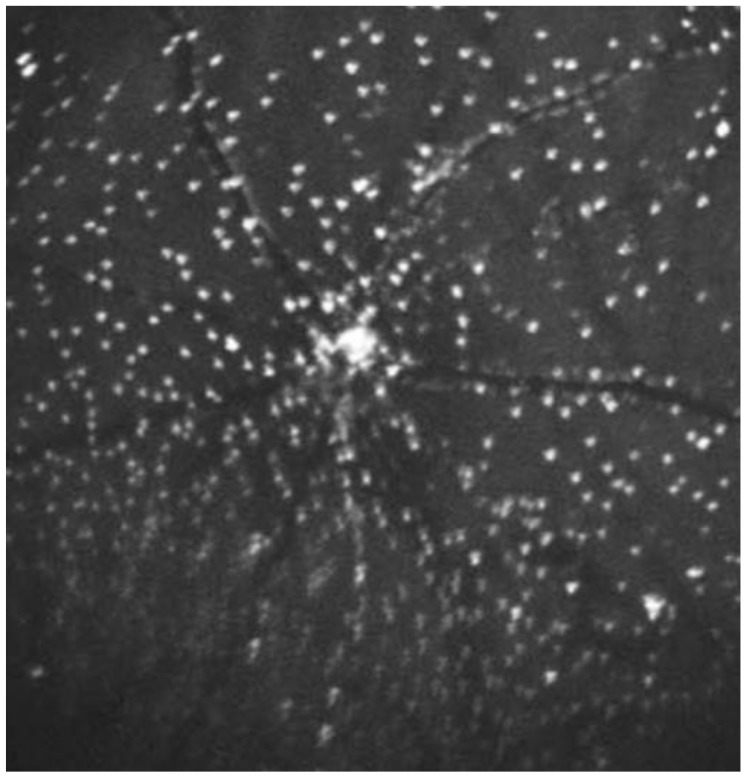
DARC imaging demonstrating the detection of fluorescently-labelled annexin A5 molecules in apoptosing retinal ganglion cells in a rat model of ocular hypertension [102]. The model is formed using the injections of hypertonic saline into episcleral veins. Fluorescent markers have been introduced by intravitreal injections of ANX776 and imaged using confocal scanning laser ophthalmoscopy. The confluent staining at the optic nerve head is as yet of unknown significance, but may represent axonal staining as the nerve fibers enter the optic disc.

**Figure 5:**
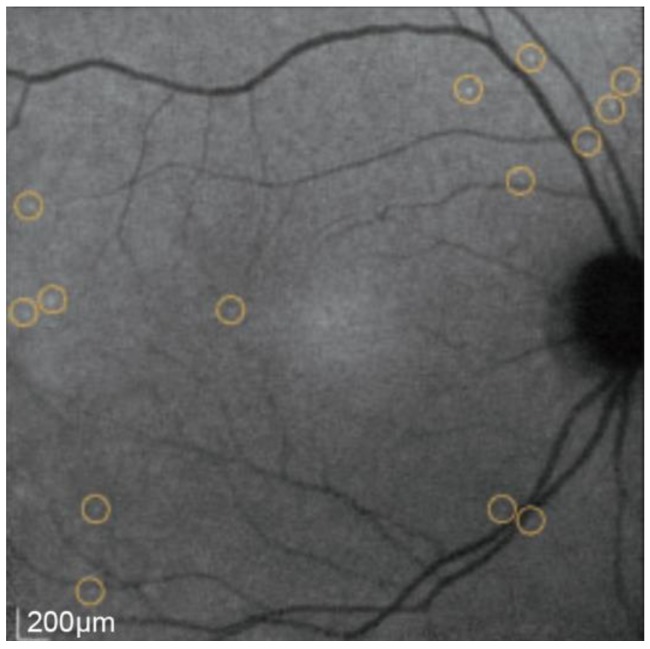
DARC imaging showing the circled apoptosing RGCs following intravenous injection of ANX776 in a human retina.

As the technique has developed, an average of 100 frames has been introduced in combination with eye-tracking to achieve a higher signal-to-noise ratio. After this, the images undergo several transformations to compensate for non-linear optical distortions and large non-enhancing structures, such as the blood vessels [87,88,89]. Analyzing these images produces a “DARC count” using a template-matching approach [90] to detect the number of annexin A5-labelled spots. These spots are seen as hyperfluorescent spots measuring between 12 and 16 μm in diameter (Figure 4 and Figure 5).

Histological evidence supporting DARC spots as apoptosing RGCs has been demonstrated using the dual-labelling of annexin 5 and caspase-3 positive RGCs. This was accomplished using the retrograde labelling of RGCs by an injection of DiAsp (4-(4-(didecylamino)styryl)-*N*-methylpyridinium iodide) to the superior colliculi of rats. This was combined with histological staining using 4,6-diamidino-2-phenylindole (DAPI) to assess the nuclei, Cy5-labeled anti-caspase-3 to confirm apoptosis and fluorescent-labeled annexin A5 to represent the DARC spots in vivo [91].

Since the DARC technique was established in 2004 [91], various experimental animal models have been assessed. This includes testing the efficacy of neuroprotective agents and the characterization of the natural history of experimental glaucoma in addition to investigating other disease models, including Alzheimer’s disease (AD) [92,93,94] and Parkinson’s disease (PD) [95,96,97,98,99,100]. Glaucoma-related models can be induced by either surgically or chemically causing RGC apoptosis [101]. To evaluate DARC sensitivity and accuracy in monitoring RGC apoptosis, its performance was first assessed in the well-established rat models of chronic ocular hypertension (OHT) and optic nerve transaction (ONT) [91]. RGC apoptosis was demonstrated to occur over time, peaking at 3 weeks in OHT and 7 days in ONT models. At the end of the experiments, RGC apoptosis accounted for a total RGC loss of 60% at 16 weeks in OHT and 76% at 12 days in ONT models. RGC apoptosis was also induced in rodent models by intravitreal administration of staurosporine (SSP) [86] and dimethyl sulfoxide (DMSO).

Glutamate excitotoxicity has been implicated in the death of RGCs in glaucoma [23,24,103]. Blocking the *N*-methyl-d-aspartate (NMDA) receptors with specific antagonists, such as memantine, was reported to be effective in reducing RGC death in experimental glaucoma [104,105,106] (a non-competitive NMDA receptor antagonist; the best-known glutamate modifier in Alzheimer’s disease treatment [107]). DARC has been used to assess the effects of different glutamate modulation strategies, including non-selective (MK801) and selective (ifenprodil) NMDA receptor antagonists, and a metabotropic glutamate receptor agonist (mGluR Group II, LY354740) in an SSP-induced rat model of RGC apoptosis [98]. All three single agents significantly reduced RGC apoptosis in a dose-dependent manner but combining low-dose MK801 with LY354740 appeared to be most effective compared to either agent alone. An optimal combination regimen was then applied to an OHT model at different time points (0, 1 and 2 weeks following IOP elevation). DARC revealed that the most effective timing of treatment was at the time of IOP elevation. This was attributed to the maximal inhibition of glutamate release occurring after the primary insult, thereby only modifying the secondary degeneration processes [108].

Another pathological process investigated with DARC is the deposition of amyloid-β (Aβ) plaques, which are characteristic of Alzheimer’s disease. Aβ deposition is also implicated in the development of RGC apoptosis in glaucoma [99,109,110]. Aβ is derived from the proteolytic cleavage of amyloid precursor protein (APP). Two catabolic pathways are identified for APP processing: one is the non-amyloidogenic pathway via α-secretase activation, resulting in secretion of soluble forms of APP (sAPPα) and the other is the amyloidogenic pathway, where β- and γ-secretase activation leads to Aβ generation. DARC has been used to assess the effects of targeting the amyloidogenic pathway in an OHT rat model by examining three different agents: an anti-Aβ antibody (Aβab), a β-secretase inhibitor (βSI) and Congo red (CR). CR is a dye commonly used to stain amyloid-β histologically and has been shown to block Aβ aggregation [111,112]. DARC data showed that all three single agents altered the profile of RGC apoptosis in a temporal manner by delaying the peak RGC apoptosis and reducing peak levels. Although the anti-Aβ antibodies appeared to be more effective in the prevention of RGC apoptosis than the other two agents, the combination of three agents was demonstrated to result in the maximal reduction of RGC apoptosis [99]. Modulation of the non-amyloidogenic pathway has also been investigated in the OHT rat model. Brimonidine (BMD) and clonidine (Clo) are α2 adrenergic receptor agonists (α2ARAs) drugs that are used to lower intraocular pressure (IOP) in glaucoma patients [113]. Both BMD and Clo have been reported to be neuroprotective [28,114,115]. Recently, the systemic administration of α2ARAs was found to significantly reduce the OHT-induced RGC apoptosis in vivo and was associated with reduced levels of Aβ deposition in RGC layers compared to controls. α2ARAs were also found to modulate the levels of laminin and MMP-9 (metalloproteinase-9), which are potentially linked to changes in Aβ through APP processing as they promote the non-amyloidogenic pathway [116].

The pathological process behind Parkinson’s disease (PD) has also been studied using DARC, the hallmark of which is the death of dopaminergic cells in the substantia nigra [117]. Visual phenomena are also found in PD, including reduced visual acuity, color vision and contrast sensitivity [118,119]. For this study, a rotenone-induced rodent model of PD [120] was imaged to test the effects of rosiglitazone, which is a peroxisome proliferator-activated receptor gamma (PPAR-γ) agonist [95]. Using DARC, the results demonstrated that RGC apoptosis peaked at 20 days post-rotenone, compared to the classical pathological findings in the substantia nigra, which were present at day 60. Additionally, intraperitoneal liposome-encapsulated rosiglitazone was shown to offer a significant neuroprotective effect in the eye at day 20 and for the changes in the brain at day 60, when compared with vehicle-only controls. These findings suggest that rosiglitazone has potential in treating PD, with a possible opportunity for the use of DARC to support early diagnosis.

Mitochondrial dysfunction and oxidative stress are known mediators of RGC death in glaucoma [121,122]. Coenzyme Q10 (CoQ10) is an antioxidant with an important role in the normal functioning of the mitochondrial electron transport chain. CoQ10 exhibits neuroprotection in neurological disorders, such as AD, PD and Huntington’s disease [122], as well as in experimental glaucoma [123]. DARC has been used recently to evaluate the topical administration of CoQ10 formed into micelles using the vehicle tocopherol polyethylene glycol succinate (TPGS) in an OHT rat model [102] (see Figure 4). DARC data demonstrated that topical CoQ10 treatment significantly reduced the OHT-induced RGC apoptosis compared to vehicle-only controls. DARC results are consistent with the retinal wholemount histology outcomes, in which topical CoQ10/TPGS (but not TPGS treatment) can protect Brn3a+ RGCs against apoptosis as indicated by the preservation in RGC density and nearest neighbor distance.

Cell-based therapies are becoming an increasingly recognized strategy with the potential to treat retinal neurodegenerative disease [124,125]. Unfortunately, in their current experimental settings, complex and indirect administration techniques are involved, with real-time monitoring of their integration into the host and effects on cell death often not possible. The unique, real-time nature of DARC allows investigators to assess the levels of apoptosis longitudinally over multiple time points in single subjects. This enabled the technique to be used to monitor RGC apoptosis and the effect of potential neuroprotective strategies over time following the direct optic nerve sheath (DONS) application of Schwann cells (SC) in a partial optic nerve transection (pONT) rat model [126]. DARC data showed that the DONS application of Schwann cells significantly reduced the pONT-induced RGC apoptosis at 7 and 21 days following pONT and SC/DONS application compared to untreated controls. The in vivo DARC results were supported by histological findings, in which SC/DONS therapy significantly increased Brn3a+ RGC survival in retinal wholemounts mostly by targeting secondary degeneration.

## 4. Annexin 5 as a Marker of Apoptosis in the Human Retina

The DARC technique has also been trialed in humans using intravenous injections of ANX776. Intravenous administration of the agents used to augment imaging is already a well-established routine in ophthalmology, which is commonly used in medical retinal clinics in the form of fundus fluorescein and ICG angiography [127]. Phase I DARC trials assessed the safety, tolerability and efficacy of detecting apoptosing human retinal cells in vivo [85]. This study included eight glaucoma patients, who had progressing disease according to either visual field (standard automated perimetry, SAP) parameters or optic nerve head imaging using optical coherence tomography (OCT) and Heidelberg Retinal Tomography (HRT). These patients were compared to eight healthy volunteers of a similar age.

The retinal imaging in the study used confocal scanning laser ophthalmoscopy (cSLO) and fluorescent settings for ICG angiography (diode laser with 786-nm excitation and photodetector with 800-nm barrier filter). Images were captured from a 30-degree field of view, which corresponded to an average width of 8.87 ± 0.28 mm of retina. The imaging was performed at baseline and following injections of 0.1 mg, 0.2 mg, 0.4 mg and 0.5 mg of ANX776 in four groups of four patients, two healthy and two glaucomatous. Imaging was captured at 15, 30, 60, 120, 240 and 360 min timepoints post-injection (Figure 5). One patient was excluded from analysis due to abnormal baseline visual fields. The results demonstrated that the DARC count was significantly higher (*n* = 15, *p* = 0.0033, 2-way ANOVA) in the glaucomatous cohort of patients when compared to the normal subjects across all the doses. In particular, at the 0.4 mg dose, the mean DARC count was 25 in the glaucomatous group compared with 10 in the healthy controls (*n* = 4, *p* < 0.005). Other factors that were significantly associated with the DARC count were thin central corneal thickness (Spearman’s R = −0.68, *p* = 0.006) and high cup-to-disc ratio in glaucoma patients (Spearman’s R = 0.47, *p* = 0.038). Post-hoc analysis showed that the DARC count was significantly increased in glaucoma patients with an increasing rate of progression in any parameter (HRT, OCT or SAP) when compared to healthy controls (Dunn’s multiple comparison test, *p* < 0.05), although this was not significantly different from stable glaucoma patients.

With regards to safety, no patients withdrew from the study and no serious adverse events were recorded. Six mild adverse events were recorded in all 16 participants, including issues related to intravenous cannulation, headache, influenza, dizziness and metatarsal inflammation. These adverse events were deemed unlikely to be related to the ANX776 injection. The majority of the remaining complaints were symptoms that patients had previously suffered from, except one patient, who was diagnosed with metatarsal inflammation 3 weeks following the injection. The pharmacokinetics in all 16 participants were examined by serial blood tests at 5, 15, 30, 60, 120 and 300 min using 0.1, 0.2, 0.4 and 0.5 mg doses, which demonstrated fast absorption (time to maximum serum concentration, T_max_ = 5.0–7.0 min), dose-dependent maximum serum concentrations (5.5–40.9 ng/mL) and half-life (inversely related, 36.4–10.1 min) and no accumulation (minimum serum concentration, C_min_ = 0.6–1.0 ng/mL).

Phase II of the DARC project is currently investigating the efficacy of DARC in visualizing apoptosing retinal cells in patients with optic neuritis, age-related macular degeneration [128] as well as in Down’s syndrome subjects as a model of Alzheimer’s disease [99,100], glaucoma patients and age-matched healthy volunteers. This project is aiming to analyze 120 subjects in total by acquiring a single DARC image following an intravenous injection of the 0.4 mg dose of ANX776, which is the dose that was found to produce the greatest difference in DARC count between the progressing glaucoma patients and healthy subjects in phase I. The results for this are to be published soon.

## 5. Discussion

From the initial animal and human studies that we have presented, considerable evidence shows that annexin A5 can successfully be used to visualize annexin-positive retinal cells using the DARC technique. The technique has been validated histologically and experimentally in animal models. In glaucoma, RGC apoptosis has been identified in the experimental models of disease and in patients clinically suffering from progressing glaucoma. Furthermore, the experiments using DARC in animal models of other conditions, such as Alzheimer’s disease, have shown promise in using the eye as a window to the brain in order to monitor and study other neurodegenerative conditions. Moreover, DARC has great potential as an endpoint for testing new therapies in all these diseases.

Results from the Phase I clinical trial suggested that DARC could be a new surrogate marker for trialing novel drugs and neuroprotective treatment strategies in glaucoma. Using the DARC count as an objective measure of real-time retinal cell apoptosis could overcome the pitfalls associated with the currently used markers of disease. In order to determine the expected proportions in findings, the estimates of the number of apoptosing RGCs one might expect to find in the human retina in healthy and glaucomatous subjects have been made [96]. Using a rat ocular hypertension model, well-documented rates of RGC loss confirmed with DARC [129,130,131,132] have been used in a lifespan-adjusted simulation to predict RGC apoptosis during a typical disease course. These projections predict that the values will range between 22 RGCs to a peak of 416 RGCs per day (15.38% of RGCs annually) at 2 years from onset. This is significantly higher in magnitude than the 8 cells per day (0.3% of RGC apoptosis annually) expected due to normal ageing. Furthermore, the validation of phase I results has been attempted by estimating the number of apoptosing RGCs expected to be visualized by DARC. Based on 30–52% of all RGCs being captured with the 30-degree lens of the Spectralis imaging system (Heidelberg Engineering, Heidelberg, Germany), it has been shown that with an average glaucomatous RGC loss of 4% [111,133], the Phase I DARC counts for the 0.4 mg cohort are within the predicted range [85].

Currently, the intraocular pressure (IOP) is the commonest endpoint of choice to prove the efficacy of the majority of treatments for glaucoma. This is due to its ease of acquisition and fast response to therapy. However, the progression of disease can occur with adequately treated IOP and glaucoma can occur when IOP is within normal limits [134]. Given the imperfect nature of IOP as a surrogate marker for visual loss, it is difficult to predict which patients with successful IOP-lowering treatments will also benefit from reduced visual loss. This is only implied with the knowledge that IOP is a known risk factor [14].

The other gold standard in glaucoma is visual field testing, but this is associated with prolonged clinical trials, which can be very expensive in assessing new treatments, despite the introduction of new algorithms for rates of progression. Using frequent repetition and computer-aided analysis of visual field tests, recent studies have shown the ability of visual field testing to reveal significant rates of progression within a two-year period [135]. Visual field testing (SAP) is also heavily reliant on the ability of the patient to carry out the test. In those patients with advanced glaucoma, poor fixation, frailty or cognitive impairment, the results are commonly unreliable. Field tests can also be influenced by other causes of visual loss (such as cataract) and can be complex to interpret as it is often dependent on subjective measures. In the patients who can reliably carry out the test, there is thought to be a lead-time (pre-perimetric glaucoma) during which the structural damage evident on OCT occurs prior to the development of a visual field defect. This is thought to represent a loss of up to 40% of RGCs [136] due to redundancy and compensatory visual processing. However, this also commonly leads to a delay in diagnosis that is proposed to be between 8 to 10 years [137]. It is foreseeable that using the DARC technique for instantly-obtainable and objective evidence of RGC disease activity would provide a good alternative for showing proof-of-confidence evidence of the treatment efficacy in the early stages of investigations, although later clinical trials would still need gold practice endpoints.

In clinical practice, the decision as to how low an individual patient’s IOP should be to avoid progression (target IOP) is difficult to predict. Clinical measurements can vary significantly according to the time of day [138] and there is conflicting evidence regarding whether or not a variation in IOP poses a greater risk to vision [139,140]. The imaging of the retinal nerve fiber layer and ganglion cell complex is a more direct measurement of glaucomatous damage. Similar to visual field testing, the time taken to detect progression in structural parameters with accompanying visual field loss are significant disadvantages. In contrast, DARC holds potential in differentiating progressing glaucoma patients from healthy individuals without the requirement of time, which could potentially expedite diagnosis at the initial consultation [85]. An ultimate aim of DARC in glaucoma is to achieve diagnosis during the “pre-perimetric” phase, which is thought to last up to ten years from disease onset [136,137] in order to avoid significant visual loss. The non-invasive administration of DARC that is currently under development would enable it to be used as a screening tool. There is also a role for DARC to identify patients, who are unlikely to have progressive disease. Using this technique could free them from the burden and side effects of daily eye drop use and the known detrimental effect that diagnostic suspicion has on quality of life [141]. Potentially, the DARC technique could also indicate when treatment needs to be intensified in progressing patients. Furthermore, disease stability could be confirmed at a certain “target IOP”, which would especially benefit patients, who cannot afford to lose what little vision they have left whilst under monitoring for progression by traditional methods. Although ocular co-morbidities could be controlled for in the research setting, patients with conditions, such as diabetic retinopathy or age-related macular degeneration, may also exhibit a higher DARC count than healthy subjects of a similar age. Further characterization of the appearance of other conditions is taking place in phase II clinical trial of DARC and subsequent analysis is required to determine exactly how these will be differentiated.

With ageing populations and lengthening life expectancy in many countries, the number of glaucoma patients is rapidly increasing. From an estimated 64.3 million people (prevalence of 3.05%) in 2013, it is estimated that glaucoma sufferers worldwide could reach 111.8 million by 2040 [142], creating significant logistical and financial strain on healthcare systems. To ameliorate this, DARC may have a role to play in reducing the number of patients under observation due to suspicion of glaucoma brought about by the disc appearance or raised IOP in isolation. Although the DARC technique is minimally invasive, the intravenous cannulation of all patients at every subsequent visit is unlikely to be feasible or acceptable to most people. In addition, the technique is also currently limited by a specific time window and is unable to indicate the total number of RGCs remaining. However, as a role in screening, DARC could prove to be a valuable tool in the clinician’s armory in cases where progression is suspected to occur independently of IOP or when visual field testing is unreliable. The advantage of DARC in all these scenarios is that it could reduce the health and socio-economic burden by promoting earlier intervention and rapid assessment of therapy changes in those patients with active and progressing disease in addition to identifying stable, low risk patients and those with no disease activity, who could be discharged from hospital outpatients back to primary healthcare.

Further work to be done with DARC will involve both continual sophistication of the technique and its applications in humans. The technique itself has the potential to be improved in terms of sensitivity and cell-type differentiation in addition to possibly exploring alternative routes of fluorescent marker administration. New applications in humans will involve increasing patient numbers, diversifying disease pathologies under examination and testing the effects of existing and novel treatments on the levels of retinal cell apoptosis.

## Figures and Tables

**Table 1:** Summary of some disorders in which apoptosis dysregulation has been implicated.

Condition	Pathology	Reference
Glaucoma	Accelerated apoptosis of retinal ganglion cells.	[2,43,44]
Age-related macular degeneration	Accelerated apoptosis of retinal pigmented epithelium, photoreceptors, and inner nuclear layer cells. Autophagy and necrosis may also play a role.	[45,46]
Diabetic retinopathy	Accelerated apoptosis of neural and vascular cells leading to increased vascular permeability and reduced visual function.	[47]
Alzheimer’s disease	Accelerated neuronal apoptosis.	[48,49]
Huntingdon’s disease	Accelerated neuronal apoptosis.	[50,51,52]
Parkinson’s disease	Accelerated apoptosis of dopaminergic neurons.	[53,54]
Malignant gliomas	Resistance to apoptosis.	[55]
Melanoma	Resistance to apoptosis.	[56]
